# Recommendations for Assessment of Social, Emotional, and Behavioral Health for the National Children's Study

**DOI:** 10.3389/fped.2021.624524

**Published:** 2021-05-04

**Authors:** Cindy J. Nowinski, Darren A. DeWalt, Alice S. Carter, Anil Chacko, Heather E. Gross, Eliana M. Perrin, Chelsea Weaver Krug, Jane L. Holl, Richard C. Gershon

**Affiliations:** ^1^Department of Medical Social Sciences, Northwestern University Feinberg School of Medicine, Chicago, IL, United States; ^2^Cecil G. Sheps Center for Health Services Research, University of North Carolina, Chapel Hill, NC, United States; ^3^Department of Psychology, University of Massachusetts Boston, Boston, MA, United States; ^4^Department of Applied Psychology, New York University, New York, NY, United States; ^5^Department of Pediatrics, Duke University School of Medicine, Durham, NC, United States; ^6^Department of Psychology, University of Pittsburgh, Pittsburgh, PA, United States; ^7^Department of Neurology, Center for Healthcare Delivery Science and Innovation, University of Chicago, Chicago, IL, United States

**Keywords:** outcomes assessment, longitudinal research, health assessment, pediatric longitudinal research, social emotional health

## Abstract

The Social Emotional Behavioral (SEB) Team of the National Children's Study (NCS) was tasked with making recommendations for assessment of important aspects of social-emotional health and function in children. This paper describes the constructs recommended for assessment along with the rationale for their assessment. These constructs, representing aspects of Social Relationships, Social Capital, Temperament, Negative Affect, Externalizing Behavior, Social Competence, Self-efficacy, Self-image, Psychological well-being, Ethnic/racial Socialization, Perceived Discrimination, Sexual Orientation, Religiosity, and Perceived Stress and Resilience were identified as being critical to the understanding of children's health and development from birth to age 21.

## Introduction

The National Children's Study (NCS) was conceived as a national longitudinal study of the environmental factors that influence children's health and development. In 2013, the NCS established a multi-institutional, collaborative Health Measurement Network (HMN) to develop a scientifically rigorous and practically feasible framework for assessing these factors and their impact on child health and well-being (the history and purpose of the NCS are described in the Introduction to this Research Topic). The HMN consisted of six teams focused on critical domains of health, one of which was social-emotional health.

Children's social-emotional well-being affects their relationships and interactions with others, management of emotions, and ability to learn. In general, children who show positive social-emotional health are more likely to develop into adaptable, functioning adults. Developmental disruptions during the formative years can impair children's capacity for educational and future economic achievement, and optimal health later in life (1). Such health risks to our nation's children also have societal implications in that not just the individuals themselves, but their families and communities are affected. Social contexts are far-reaching determinants of development and health, and it is important to understand how children develop and adapt to their physical and social environments (2). If we can identify detrimental and beneficial influences associated with developmental milestones, mediators of social and behavioral inequities, and strategies for intervention, then parents, other adults, and communities can be effective in promoting positive social emotional health outcomes (e.g., resilience, optimism, feeling of belonging) and support aspects of social-emotional health in children and within their families (3).

### Methods

The Social Emotional Behavioral (SEB) Team of the National Children's Study Health Measurement Network was tasked with making recommendations for assessment of important aspects of social-emotional health and function in children. This work had two phases, the first identified the most critical constructs to assess and the second proposed the best options for each recommended assessment within the NCS measurement framework. To perform this work, each team member assumed primary responsibility for reviewing relevant literature, similar national and international studies and, for the second phase, databases of measures. They then presented a summary of their findings to the entire SEB team for discussion. After reaching consensus, the SEB team presented their recommendations to the entire HMN for final approval. The constructs recommended for assessment and the rationale for their assessment are the primary focus of this paper, since information about the constructs can expand our understanding of how to foster children's health and development; how best to measure each construct is highly study-dependent. Therefore, we do not provide specific measures in the text but include a table of recommended measures at the end of this paper. We remind researchers that these measure recommendations were made within the context and constraints of the NCS. The NCS measurement plan included in-person and remote assessments (typically requiring no more than 60–90 min of total time from participants) to assess multiple aspects of children's environment and their cognitive, motor, sensory, physical, and social-emotional health at specific time points in children's development. We expected this plan would have been refined through our experience over time as the Vanguard (pilot) Study and the Main Study (which never started) was implemented and through ongoing scientific and measurement advances in the study of child health. The HMN established measure selection criteria that included both scientific and practical considerations and recommendations for instruments and measures may have been different for a study, for example, with different or fewer constraints.

Scientifically, measures needed to be valid, reliable, and appropriate for use at planned assessment time points (e.g., infancy, adolescence). These criteria, set by the HMN Statistical and Item Response Theory Group, are described in the Hays et al. paper Methodological and Statistical Considerations for the National Children's Study. Once scientific criteria were met, in order to maximize feasibility, preference was given to measures that were free or low cost (e.g., non-proprietary), brief (e.g., to maximize the number of outcomes and environmental variables able to be assessed at each time point), easy to administer (e.g., did not require extensive training or education beyond a bachelor's level), and minimally burdensome to participants. These instrument characteristics are especially important for longitudinal studies like the NCS, which planned to follow 100,000 children from before birth through 21 years of age. These measures may not always be the best fit for other types of studies. The SEB team reviewed many measures identified from the literature. For most measures, only final recommendations are included in the table. These final recommendations were those that most closely met NCS criteria. Many excellent measures exist that did not fit the NCS-specific criteria and, therefore, are not included. In other cases, we included some measures that potentially could be used in the NCS, given further development. For example, observational measures are particularly useful during infancy through early childhood, when children are unable to or have difficulty responding to self-report instruments, but often require extensive behavioral coding that is impractical for a study as large as the NCS.

We also recommend “banking digital media” or investing in recording assessments and storing digital media that could be acquired longitudinally and saved for future coding. As videotaping becomes less expensive to collect and store and as artificial intelligence, machine learning, and computer scoring of video data becomes increasingly reliable (e.g., coding affect, coding of gaze), it will become more cost-effective to retrospectively analyze stored video data for the purposes of completing case-control designs for a wide variety of conditions that emerge later in childhood and early adulthood. Video and audio data capture of child and parent responses to both frustration and reward, as well as to social initiations and novelty may prove to be extremely useful for understanding early predictors of a range of psychopathological conditions that may not emerge until later in development (e.g., schizophrenia, depression, anxiety disorders). These videotapes would also be a critical resource for documenting normative developmental trajectories and the expected wide range of typical variance that characterizes different developmental periods. Moreover, we recommend videotaping and storing brief parent-child interactions. The banked parent-child video data could illuminate important components of children's environments expected to influence or predict later language, self-regulation, academic achievement, and other outcomes. These components include the number and quality of words presented to the child; use of comments, gestures, and demands; pacing, reciprocity, and synchrony of verbal, affective, and tactile behaviors in parents and children; as well as parental autonomy promotion, warmth, praise and criticism.

The cost of collecting video data and storing the digital media is offset by the potential scientific gains that can be achieved by having these data available once children begin to evidence clinically significant health conditions or as deeper understanding of specific observable behaviors relevant to supporting healthy developmental trajectories emerges.

## Results

### Social Relationships: Parents, Family, and Peers

Children develop within the context of social relationships that become increasingly complex over time, as there are complex interactions between children's individual characteristics and their social relationships. Social relationships can be characterized by their structure, quality, quantity, and other dimensions (4). They can be examined in terms of properties of the *relationship*, as well as, characteristics of the *individuals* (e.g., parent, child, peer) involved in the relationship. These relationships are also bi-directional, with parents, family, and peers (the major relationship groups for children) not only influencing child development but also being influenced by the child's characteristics.

#### Importance of Parent-Child Relationships

Parent-child relationships are typically the earliest relationships. Parent-child interactions and the quality of the parent-child relationship influence the development of emotional and social skills and competencies that, in turn, affect children's other relationships. The nature and quality of parent-child interactions have been associated with both positive and negative aspects of children's social and emotional development, including social competence (5), social-emotional adaptation to school (6), and emotional and behavioral problems and psychopathology during childhood and adolescence (7, 8). Parenting practices can also mediate other aspects of health, such as physical activity and weight status (9, 10). When assessing the parent-child relationship, the focus can be on the parent (as an exposure) or the child. Thus, quality can be considered as both an exposure and, particularly, as the child gets older, as an outcome. In contrast, while recognizing that parent-child interactions are bidirectional in nature, we consider parenting practices as an exposure that can affect child health outcomes.

Different aspects of the parent-child relationship have been studied. These include broad constructs such as *parenting style* and *positive parenting*, as well as narrower ones such as *responsiveness, discipline, nurturance, warmth, hostility*, and *control*. Parenting style conceptualizes parenting in terms of typologies that encompass a wide range of parenting behaviors, dimensions, and attitudes. Darling and Steinberg (11) define parenting style as the “constellation of attitudes toward the child that are communicated to the child and create an emotional climate within which the parent's behaviors are expressed.” Parenting style includes both what parents do (behaviors) and how they do it (quality and valence of parent-child interactions) (12). The original typologies proposed by Baumrind (13) were *authoritative, authoritarian*, and *permissive*. A fourth typology, *disengaged* parenting, has also been proposed, with these 4 typologies conceived of as varying along the dimensions of parental responsiveness and demandingness (14). We recommend assessment of parenting style, as well as, narrower dimensions of the parent-child relationship (some of which are subsumed within parenting style). This multi-level approach could enable examination of the relative contributions of, and interactions between, specific dimensions. Furthermore, research suggests that there are ethnic, racial, and cultural differences in the relationship between aspects of the parent-child relationship and child health outcomes. Assessment of multiple dimensions can therefore increase our understanding of cultural, ethnic, racial, and socioeconomic effects. We recognize that extreme forms of parenting, including child abuse, neglect and/or deprivation, are also important determinants of child health outcomes. These constructs are targeted for assessment under the Environment domain.

Currently, available measures do not provide continuous coverage of this domain from birth through age 21. Further, the frequency and salience of specific aspects of parenting and parent-child interactions change as children develop. For example, parental use of physical discipline is more common with younger children while practices around autonomy become more important during later childhood, adolescence, and early adulthood. These developmental changes likely preclude a single measure that would be relevant across all ages and even preclude linking scores in a manner that would allow for direct comparison of scores for a 3- and a 12- year old, for example. Therefore, in our Recommended Measures Table, we include several measures that each cover a different age range.

##### Family

Children's experiences of family belonging and family involvement begin early in life and become a constant and powerful influence on the willingness, motivation, and ability of children and youth to take reasonable risks, moderate their behavior, and develop effective social and emotional skills and competencies. Indeed, the need to belong is a primary human motivation that involves feeling valued and cared about by family and others who are defined as important (15). Feeling that one belongs also involves knowing that one can trust and depend on those people for support and help (16).

#### Importance of Family Belonging and Involvement

A strong sense of belonging is dependent on having frequent interactions with one's parents and family (and any other valued social group) that are perceived as accepting, supportive, and enjoyable (16, 17). We refer to these everyday verbal and behavioral interactions as “family involvement.” The child's perceptions of the quality of these interactions with family members include their perceptions of how consistently they have interactions with parents and other family members that allow them to communicate their needs and ideas and enjoy activities together.

Much evidence now documents that children with positive, secure connections to parents and family tend to have better self-regulation, social skills, and emotional and behavioral functioning, and are, thus, better able to cope with the demands of life (18). Children's sense of belonging appears to begin in infancy and early childhood, supporting Bowlby's theoretic position that early affiliative bonds between infants and their parents shape future relationships with family, as well as peers (17). Compelling longitudinal research provides evidence that the security of children's connections to their parents and family has a powerful influence on their development (19) and on their emotional and behavioral functioning (18). Disruptions in early parental care exert profound and lasting impacts on brain development, gene expression, and vulnerability to later stress, anxiety, and affective disorders (20–22). Berkman et al. (23) developed a model that describes how social connections influence psychobiological processes and ultimately health, disease, and mortality across the lifespan.

Children's experience of family relationships involves both the affective or emotional feelings and beliefs about family belonging, as well as the behavioral or family involvement component by which the sense of belonging is maintained. In a population of children, belonging and involvement are expected to range from very positive to very negative. Measuring the full continuum of family experience from multiple perspectives (e.g., parent and child) will facilitate a greater understanding of normative and dysfunctional family development and the family's contribution to children's health.

##### Peers

Peer relationships refer to relationships with friends and other acquaintances. These interactions are multileveled (individuals, relationships, and groups) and are quite socially complex. Peer relationships are affected by children's other relationships (e.g., those with their parents, siblings, and teachers), the quality of their parents' own relationships, and their own style of interacting [e.g., degree of prosocial behavior, aggressiveness, or behavioral inhibition/shyness they exhibit; (24)].

#### Importance of Peer Relationships

Peer experiences affect social, psychological, and physical health and development, both positively and negatively (25–27). Bidirectional effects occur between peer relationships and individual characteristics, including cognitive and emotional processes such as empathy, emotionality, and attentional and emotional regulation (28, 29). Peer relationships can be evaluated in terms of how children behave with peers (e.g., prosocial, aggressive, impulsive behaviors), the quality of their peer relationships (e.g., acceptance, rejection, supportive, friendship), and children's role within the relationship (e.g., leader, bully, victim).

Assessment in early childhood, which typically relies on parent report, usually focuses on “behavior” toward peers, since proxy report of observable behaviors tends to be more reliable than proxy report of subjective experiences. In later childhood through young adulthood, we believe that self-report is a more accurate reflection of peer relationships than parent report, and have recommended measures as such. Measurement of peer report could expand the understanding of peer relationships, but was not planned for the NCS for feasibility reasons.

### Social Capital

There is no consensus on the definition or measurement of social capital, although Whitley and McKenzie (30) concluded that networks, relationships, norms, and trust seem to be basic components of most definitions of social capital. Some theorists see social capital as a property of individuals while others see it as a property of an ecological unit (e.g., neighborhood). Individual-level social capital is conceptually overlapping with social support/social network theory. Additional conceptualizations of social capital (e.g., bridging, bonding, cognitive, structural) add even more theoretical complexity.

#### Importance of Social Capital

Social capital at the individual level (e.g., social support) has been shown to act as a protective factor for a wide variety of health outcomes including mortality, accidents, suicides, cardiovascular disease, alcohol use, substance abuse, and depression (30–33). At the community level, social capital has been shown to have protective effects on child and adolescent health outcomes including self-rated health, physical health complaints, and health behavior (34). Further, community-level social capital and the components of bonding, bridging, and linking have been shown to mediate the effects of socioeconomic inequalities on health (34, 35). In addition to mediating direct effects, social capital also seems to interact with socioeconomic status, having a stronger positive effect on health for people of lower SES (35).

Although social capital has been defined and measured at the individual and community level, we recommend assessment of social capital at the community level, in association with child health and development, and an assessment of social support/social network in lieu of individual social capital in the parents.

### Temperament

Temperament refers to a set of emotional and behavioral repertoires that reflect individual differences. Temperament is established early in life and forms the basis for later adult personality. Temperament is considered to be biologically and genetically based, but can be modified by environmental influences.

#### Importance of Temperament

Temperament has been linked to a wide range of social, emotional, and behavioral outcomes, including empathy, conscience, guilt, internalizing and externalizing behavior, and development of psychopathology (36–39). Temperament has also been linked to other aspects of physical health. After a recent review of the literature, Anzman-Frasca et al. (40) concluded that greater levels of negative reactivity in early life appear to increase the risk of obesity, while greater self-regulation may be protective.

We recommend early observational assessment of temperament.

### Emotional Distress/Negative Affect: Anger, Depression, and Anxiety

Emotion is an affective state of consciousness in which joy, sorrow, fear, hate, or the like is experienced, as distinct from cognitive and volitional states of consciousness. The experience of emotion varies along dimensions of frequency, intensity (arousal), valence (positive or negative), and lability (degree of fluctuation in mood state). Studies indicate that in community samples, normative daily experience involves emotions of low to moderate intensity (41). High “emotionality,” characterized by frequent intense affective reactions is unusual. Indeed, research suggests that individuals appear to have trait-like tendencies to experience negative and positive emotions at a certain level of frequency and lability/variability. We recommend assessing emotions as traits, rather than transitory moods or states. Also, it is important to note that the absence of positive feelings is independent of the presence of negative feelings. Thus, any thorough assessment of emotion requires attention to the effects of positive affect, both independent of and together with negative affect.

“Negative affect” is a phrase used to describe unpleasant feelings or emotions. Such emotional distress typically is comprised of aspects of anxiety, depression, and anger, including worry, powerlessness, sadness, fear, panic, irritation, and fury (42, 43). The constructs of anger, depression, and anxiety are correlated with one another but do not completely overlap (42, 43), suggesting unique but related constructs that deserve separate attention in an assessment strategy.

#### Anger

Anger has three distinct components: Anger as an emotion, aggression as a behavioral component, and hostility as a trait reflecting the presence of cynical attitudes and mistrust of others and their motives (44). Individuals also display characteristic styles of behavioral response while experiencing the emotion of anger.

##### Importance of Anger

The emotion of anger plays an important role in theories of emotion, explanations of aggression, and treatment of psychiatric disorders (45). Disorders of anger expression and modulation are addressed in the section on externalizing behavior disorders below.

We recommend assessment of anger as an emotion and, also, as a behavior (discussed under externalizing behavior).

#### Depression

Depression can be modeled as a continuum from none or low symptoms to severe symptoms. Symptoms are primarily affective, cognitive, and somatic, and include negative mood (e.g., sadness), decrease in positive affect (e.g., loss of interest), negative views of the self (e.g., worthlessness, low self-esteem), and negative social cognition (e.g., loneliness, interpersonal alienation).

##### Importance of Depression

Depression is a common and persistent illness in youth, affecting 0.3–2.1% of preschoolers, 2% of elementary school-age children, and 5–10% of adolescents (46, 47). Females, at all ages, tend to experience greater rates of depressive disorders than males (48). Earlier age of depression onset is associated with greater psychological problems; and adolescent depression has been associated with parental behaviors that reinforce dysphoric behavior, reciprocate aggression, or fail to respond positively to children's positive behavior (8). During childhood, negative affect and internalizing behavior of children was associated with maternal depression (49).

Manifestations of negative affect vary during the lifespan. Parents can reliably report manifestations of depression and anxiety in their children at age 18 months. Therefore, we recommend parent proxy assessment in the early years. We recommend self-report starting at age 8.

Currently available measures do not provide continuous coverage of this domain from birth through age 21. As such, we pieced together different measures to cover different age ranges. We recommend trying to identify core items that are the same or similar enough for potential linking from one questionnaire to the next, over the age ranges.

#### Anxiety

Anxiety can be modeled as a continuum from none or low symptoms to severe symptoms. Symptoms include fear (e.g., fearfulness), anxious misery (e.g., worry), and hyperarousal (e.g., nervousness).

##### Importance of Anxiety

Anxiety disorders are the most common mental disorder in the general population of children and adolescents. Approximately 20% of youth experience one of the anxiety disorders and one-half as many have impairment in functioning resulting from anxiety (50–53). Retrospective reports of adults with anxiety disorders suggest that onset often occurs in childhood or adolescence (54).

Manifestations of negative affect vary during the lifespan. Parents can reliably report manifestations of depression and anxiety in their children as early as age 18 months; therefore, we recommend proxy assessment beginning at 18 months and through early childhood. Self-reported measure should begin at age 8.

As with depression, currently available measures do not provide continuous coverage of this domain from birth through age 21. As such, we recommend piecing together different measures to cover different age ranges. We encourage future efforts to identify core items that are the same or similar for potential linking from one questionnaire to the next over age ranges.

### Externalizing Behavior: Bullying and Aggression and Disruptive Behavior, Impulsivity, and Substance Use

In the child psychology and psychiatry literature, there is often a distinction made between “internalizing” and “externalizing” disorders (55, 56). While the former tend to refer to pathology of a child's thoughts, anxious and depressive moods, and internal environment, the latter construct refers to disturbed outward, distractive, disruptive, and/or destructive behaviors (57) as well as elevated angry affect and difficulties processing others' emotions [i.e., deficits in empathy; (58)]. Externalizing behaviors may include bullying, aggressive/destructive, disruptive, and antisocial behaviors. While this dichotomy has heuristic appeal, the distinction is not necessarily valid, as maladaptive manifestations of internalizing and externalizing emotions and behaviors often co-occur (59). Individuals who exhibit outwardly directed disruptive behavior, for example, may also experience internal difficulties with mood.

#### Bullying

Bullying is a subtype of aggressive behavior, in which an individual or a group of individuals repeatedly attacks, humiliates, and/or excludes a relatively powerless person (60). Bullying behaviors can be physical (hitting, kicking, pinching, taking money, or belongings), verbal (name calling, teasing, taunting, threatening), or relational (e.g., the hurtful manipulation of peer relationships that inflicts harm on others; behaviors include social exclusion and spreading rumors). Bullying can occur in a variety of contexts, including through electronic communications, which is typically referred to as cyber-bullying.

##### Importance of Bullying

A sizable number of school students are involved in peer-to-peer bullying either as perpetrators, victims, or as both. In the World Health Organization's Health Behavior in School-Aged Children survey (61), which surveyed over 200,000 11–15 year old students in 40 countries (including the United States, Canada, and numerous European and Baltic countries), the average prevalence of bullying victims and perpetrators were 12.6 and 10.7%, respectively. Further, rates of self-reported bullying were significantly higher for boys than for girls in all 40 countries (62). In terms of outcomes, a longitudinal study investigating adult psychiatric outcomes of individuals (*N* = 1420) who bullied and were bullied by peers during childhood and adolescence found that after controlling for childhood psychiatric disorders, bullying victims were more likely to have generalized anxiety disorder, agoraphobia, and panic disorder (63). Those who were bullied and who bullied others were at increased risk for depression, suicidality (males only), and agoraphobia (females only); bullies were at a significantly higher risk for antisocial personality disorder (63).

#### Aggression and Disruptive Behavior

Disruptive behaviors are a constellation of overt behavior problems that are disruptive to others and interfere with school performance, family and peer relationships, and occupational functioning (64). Examples of disruptive behaviors include physical aggression, temper tantrums, excessive argumentativeness, stealing, and other forms of defiance or resistance to authority (64).

##### Importance of Aggressive and Disruptive Behaviors

The presence of serious disruptive behaviors such as verbal or physical aggression and/or destructiveness are primary diagnostic criterion for several disruptive, impulse-control, and conduct disorders in the Diagnostic and Statistical Manual of Mental Disorders, 5th Edition (65). Further, physical aggression in children constitutes a public health problem, with victims of aggression suffering physical and mental health problems, and aggressive children showing a higher likelihood to commit violent crimes when they get older (66).

The assessment of relational aggression was considered, but due to pragmatic assessment considerations, the team decided to exclude this aspect of aggression from our recommended measurement protocol. The literature clearly suggests that the most reliable and valid measurement methods are peer reports/sociometric ratings (67–69), which would not have been feasible for the NCS.

#### Impulsivity

Impulsivity is classically thought of as acting without thinking, but it has been conceptualized as having personality (trait-like), behavioral (e.g., disinhibition), and/or cognitive (e.g., rapid cognitive tempo) components (70). Further, evidence from the neuropsychological literature suggests that behavioral impulsivity is unique from cognitive impulsivity (71). The developmental literature also suggests a distinct difference between cognitive impulsivity and behavioral impulsivity in terms of predicted outcomes (70), and impulsivity has been found to be distinct from effortful control (72).

##### Importance of Impulsivity

Hyperactivity and impulsivity, which go hand in hand, have been linked to conduct problems, academic underachievement, and impaired social competence (73, 74). Additionally, observations and ratings of impulse control among preschoolers have been shown to predict later peer rejection and deviant social problem-solving (73). Importantly, behavioral impulsivity has been shown to be more strongly predictive than cognitive impulsivity of delinquency among preadolescent boys (70).

Thus, we recommend measurement of behavioral impulsivity in addition to cognitive components of impulsivity.

#### Substance Use

Substance use is the intentional consumption of alcohol, drugs, or other substances (e.g., sprays, glues). Substance use is particularly problematic (disordered) when the recurrent use of these substances causes clinically and functionally significant impairment, such as health problems, disability, and failure to meet major responsibilities at work, school, or home. Substance use disorder is based on evidence of impaired control, social impairment, risky use, and pharmacological criteria.

##### Importance of Substance Use

The use, particularly abuse, of substances can result in significant cognitive, social, and physical impairment for the individual, as well as longer-term effects on brain development, increased mental health illness, lowered school/occupational achievement, and increased risk for accidents (75–78).

### Social Competence

Social competence refers to effectiveness in developmentally appropriate social interactions (79). Social competence can be seen as a reflection of a variety of skills, such as cooperation, helpfulness, and conflict resolution, that have age-appropriate manifestations at different developmental stages (80, 81) or, more broadly, as the flexible application of available behavioral, cognitive, and affective resources in order to achieve social goals in salient social contexts (82).

#### Importance of Social Competence

Higher levels of social competence have been associated with a variety of outcomes including better academic performance (83) and physical health (84, 85), greater peer acceptance and adoption of positive interpersonal roles (86, 87), and decreased risk of developing internalizing and externalizing behavior problems (88, 89).

#### Empathy

Empathy, one component of social competence, is the ability to perceive, understand, and share the feelings of another. It has both cognitive and affective aspects, including the ability to identify and understand another's emotions, to have an affective response to those emotions, and the ability to regulate this response. At age two, typically developing children display cognitive empathy, i.e., the ability to understand another person's intentions, and independently form their own intentions. At about age four, they have the ability to understand that another person is thinking about something in a flawed manner (90).

##### Importance of Empathy

Empathy may be foundational to the ability to care for others, as well as, a precursor to the development of prosocial behaviors, morality, and inhibition of aggression (91) and violence. Deficits in empathy have been associated with a variety of clinical disorders including sociopathy, schizophrenia, autism, conduct disorder, and disruptive behavior disorder (92, 93).

### Self-Efficacy/Locus of Control/Self-Esteem

Self-efficacy was originally defined by Bandura (94) as a personal judgment of “how well one can execute courses of action required to deal with prospective situations.” It is often referred to as the belief in one's capabilities to complete tasks and reach goals (95, 96). It is not necessarily a belief in producing a *specific* outcome following a certain behavior or action, but rather a general sense of confidence in one's own abilities to perform that certain behavior (95). Locus of control (LOC), a related, but separate, construct, is the extent to which people believe their own behavior matters in achievement or receiving a reward (97). A belief that an outcome is reliant on one's own behaviors and actions corresponds to an internal locus of control, while a belief that an outcome is affected by external forces and is not entirely dependent on one's own actions corresponds to an external locus of control (97). Internal and external LOC are not opposite ends of a single scale—it is possible to have high internal and external locus of control. Finally, self-esteem is a related, but also separate concept and refers to a person's overall evaluation of his or her own worth (98).

#### Importance of Self-Efficacy, Locus of Control, and Self-Esteem

Those with high self-efficacy, locus of control, and self-esteem are less vulnerable to external stressors or challenges and apply positivity to situations. They are more likely to have school success, set goals, and change health habits. Self-efficacy is largely positive in outcomes, with health, educational, and personal outcomes generally better among individuals with higher self-efficacy (96, 99). Social cognitive theory, originally described by Bandura (100), suggests that improvements in self-efficacy (or the belief in one's capabilities, which many interchange with the concept of confidence) are associated with positive behavior change. In terms of locus of control, some literature suggests that maternal locus of control may moderate the association between children's risky behavior and child injury rate, where external locus of control was associated with increased child injury rate for high risk children (101). In terms of self-esteem, numerous studies relate self-esteem to health and social outcomes, citing positive self-esteem as an important protective factor and negative self-esteem as a risk factor (98, 102, 103).

### Self-Image/Self-Concept: Academic Self-concept and Body Image

Self-concept is broadly defined as an individual's perceptions of him/herself, formed through experience with and interpretation of one's environment (104, 105). Early research tended to define self-concept globally, as a unidimensional construct without readily identifiable factors (104). Over the past 30 years, researchers have shifted to the understanding that self-concept is multi-dimensional (106, 107). For example, self-efficacy is a perception about oneself and therefore contributes to self-concept.

Empirical studies have found support for specific components of self-concept, including: academic ability (with separate factors for math and verbal); physical ability; physical appearance; peer relationships; and parent relationships (104, 107, 108). Social self-concept will be assessed via social relationships. Amongst the remaining aspects of self-concept, we have chosen to focus on self-concept in the areas of academic skills and physical appearance (i.e., body image). Both academic self-concept and body image have clear linkages to both positive and negative health outcomes for children, are tied to external indicators of competence (which are increasingly correlated with self-concept), and can be assessed across much of children's life course.

#### Academic Self-Concept

It is most frequently assessed in relation to academic achievement outcomes.

##### Importance of Academic Self-Concept

Numerous studies have found strong, positive relationships between academic self-concept and achievement, even after controlling for prior levels of achievement (109, 110). Academic self-concept is highly related to concurrent and previous academic achievement (109): In reciprocal-effects models, prior self-concept affects subsequent achievement, *and* prior achievement affects subsequent self-concept (111–113). Understanding the relationship between perception of self as a learner and academic achievement has important implications for both theories of learning and the selection of educational programing (112). We propose measuring academic self-concept starting at age 5. Prior research has successfully measured academic self-concept in 4 and 5 year old children, with preschoolers' reports of self-concept showing moderate correlation with academic achievement (106, 114).

Although we felt this is an important area to measure, we were not satisfied with existing measures. The team recommends updating an existing measure (see Table 1) that was developed over 30 years ago.

**Table 1:** Recommended constructs and measures for social-emotional-behavioral domain.

**Construct**	**Measure**	Type^*^	**Admin time (mins)**	**Training required**	**8 m**	**14 m**	**21 m**	**3 y**	**4 y**	**5 y**	**7 y**	**9 y**	**11 y**	**13 y**	**15 y**	**17 y**	**19 y**
Social relationships: parents	Nursing Child Assessment Satellite Training (NCAST) Teaching Scale (NCATS)	Obs	6	X	X	X	X										
Social relationships: parents	Parenting Styles and Dimensions Questionnaire (PSDQ)	PR	6.5				X	X		X		X		X		X	X
Social relationships: Parents, family, and peers	Berkeley Puppet Interview (Warmth and Enjoyment, Anger and Hostility, Responsive, Emotional Availability)	SR	16	X							X						
Social relationships: family	PROMIS Family Relationships 8-item Short Form (SF-8)	PR and SR	1.5						X-PR			X	X	X		X	
Social relationships: peers	Infant Toddler Social Emotional Assessment (ITSEA): Pro-Social Peer Interaction Scale	PR	1			X	X	X									
Social relationships: peers	NIH Toolbox Parent Report Measures of Social Relationships (17 items total)	PR	4					X		X	X						
Social relationships: peers	NIH Toolbox Self Report Measures of Social Relationships (24 items total)	SR	5									X	X	X	X	X	X
Social relationships: peers	Berkeley Puppet Interview (Peer Acceptance and Rejection, Antisocial with Peers, Prosocial Behaviors)	SR	12	X							X						
Social capital	Social Cohesion Indicators in Flanders (SCIF; 5 items)	PR and SR	1			X-PR		X-PR				X	X	X		X	
Temperament	Rothbart Infant Behavior Questionnaire-R: Activity Level (7), Distress to Limitations (8), Fear (7), Duration of Orienting (6), Smiling and Laughter (7), Soothability (7), Falling Reactivity (7), Perceptual Sensitivity (6), and Approach (6) for a total of 61 items	PR	12		X	X											
Temperament	Rothbart Early Childhood Behavior Questionnaire-R Effortful Control: Inhibitory Control (6), Attention Shifting (8), Attention Focusing (6), Low-intensity Pleasure (6) and Cuddliness (6) for a total of 32 items	PR	6				X	X									
Temperament	Laboratory Temperament Assessment Battery (Lab-TAB; select presses)	Obs	12	X	X	X		X									
Anger	NIH Toolbox Proxy Anger 3–7 (9 items)	PR	1.5						X	X							
Anger	NIH Toolbox Self-Report Anger (Same as PROMIS Pediatrics Anger self-report)	SR	1.5									X				X	
Depression	BITSEA parent report	PR	2				X	X									
Depression	Berkeley Puppet Interview (Depression)	SR	4	X					X								
Depression	NIH Toolbox Sadness 3–7 Parent Proxy Report	PR	1.5							X	X						
Depression	Child Self-Report PROMIS Depression	SR	1.5									X	X	X		X	
Anxiety	BITSEA parent report (internalizing)	PR	1.5				X	X									
Anxiety	NIH Toolbox Fear 3–7 Parent Proxy Report	PR	2					X		X							
Anxiety	Berkeley Puppet Interview (Overanxiousness)	SR	4	X							X						
Anxiety	Child Self-Report PROMIS Anxiety (same as NIH Toolbox Child Self-Report Fear)	SR	1.5									X	X	X		X	X
Bullying	The Youth Risk Behavior Surveillance System (YRBSS; 2-item self-report)	SR	0.5									X	X	X	X	X	
Aggression and disruptive behavior	Multidimensional Assessment of Preschool Disruptive Behavior (MAP-DB) 10 item subset	PR	2				X	X	X	X							
Aggression and disruptive behavior	Behavior Problems Index (BPI); 10-item CBCL-based Externalizing Scale	PR and SR	2							X-PR	X-PR	X-PR	X-PR	X-PR		X	
Impulsivity	Infant-Toddler Social and Emotional Assessment (ITSEA; Activity/Impulsivity)	PR	1				X	X									
Impulsivity	Disruptive Behavior Disorder Rating Scale (DBD)	PR	2								X	X		X			
Social competence	Social Competence and Behavior Evaluation Inventory-30 (SCBE-30)- Social Competence Scale	PR	2					X		X	X						
Social competence-empathy	NIH Toolbox Empathic Behaviors	PR	2					X		X							
Social competence-empathy	Empathy scale from the Infant-Toddler Social and Emotional Assessment	PR	1.5					X		X							
Self-efficacy/Locus of control/Agency	NIH Toolbox Self-Efficacy Survey	SR	2										X			X	
Self-image/Self-concept: academic self-concept and body image	Adaptation of the Pictorial Scale of Perceived Competence and Social Acceptance for Young Children (PSPCSA)	SR	TBD							X							
Self-image/Self-concept: academic self-concept and body image	CHIP-CE/Healthy Pathways Achievement Measure (5 items)	SR	1									X		X		X	
Body image	Body-Esteem Scale for Adolescents and Adults (BESAA)	SR	2									X		X		X	X
Positive affect	PROMIS Pediatric Positive Affect	PR and SR	1							X-PR		X	X	X		X	X
Life satisfaction	PROMIS Pediatric SF-4 Life Satisfaction—Life Satisfaction	SR	1									X	X	X		X	X
Purpose in life	PROMIS Meaning and Purpose SF-4 Meaning and Purpose in Life	SR	1									X	X	X		X	X
Optimism	Youth Life Orientation Test (YLOT)	SR	4									X	X	X		X	X
Ethnic-racial socialization	Ethnicity typically emphasizes the common history, nationality, geography, language, food, or dress of groups of people (such as Haitian, African-American, European-American, Dominican, Irish, Cantonese, etc.). In your own words, to which ethnic group(s) do you belong?”	PR and SR	10							X-PR		X-PR			X	X	X
Ethnic-racial socialization	Ethnic-Racial Socialization-cultural socialization subscale	PR	1							X		X					
Ethnic-racial socialization	Multigroup Ethnic Identity Measure-Revised (MEIM-R)	SR	1												X	X	X
Perceived discrimination	The Everyday Discrimination Scale Short Version	SR	1									X				X	
Childhood gender and non-conformity	Development of an abbreviated Pre-School Activities Inventory (PSAI)	PR	TBD^**^				X-PR	X		X							
Attraction, identity, and behavior	Sexual Attraction Questionnaire	SR	TBD													X	
Attraction, identity, and behavior	Gender Identity Question	SR	TBD													X	
Attraction, identity, and behavior	Sexual Activity Questions	SR	TBD													X	
Religiosity	DUKE University Religion Index (DUREL)	SR	1										X	X		X	
Perceived stress and sources of resilience	PROMIS Pediatric Stress Response, Psychological Stress Experiences Short Form-4	SR	1										X	X		X	X
Resilience	PROMIS Pediatric Stress Response, Psychological Stress Experiences Short Form-4 -Resilience Subscale	SR	1								X				X	X	X
Resilience	Resilience Scale for Adolescents (READ)	SR	TBD												X	X	X

*PR, parent report*.

* *Obs, observational; PR, parent-report; SR, self-report*.

** *TBD, Measure to be developed or adapted, so admin time unknown*.

#### Body Image

Body image is broadly defined as an individual's perceptions of his/her physical appearance. Body dissatisfaction is related to maladaptive eating attitudes and behaviors (e.g., dieting, exercising to lose weight) in children (115, 116).

##### Importance of Body Image

When these behaviors are present in childhood, individuals are at long-term risk for chronic body image problems, weight cycling, obesity, and eating disorders (117). Body dissatisfaction in adolescence is also associated with depression for girls and engaging in risky behaviors, such as anabolic steroid use, for boys (118).

### Psychological Well-being: Positive Affect, Life Satisfaction, Meaning and Purpose, and Optimism

Psychological well-being includes both hedonic and eudaimonic components (119, 120). Hedonic components are more subjective and experiential and emphasize pleasure and positive emotions and moods associated with happiness, serenity, and cognitive engagement. Eudaimonic well-being is more evaluative in nature (e.g., meaning, optimism, life satisfaction). Positive affect has been characterized as happiness, contentment, high energy, and interest (121), and it includes activated/excited and non-activated/peaceful components. It occurs both as a range of positive emotions that occur sporadically as well as a stable and consistent trait that is inherent in one's personality. Life satisfaction, a central component of well-being, is the assessment of one's life and life's conditions, relative to one's expectations and in comparison with others. Life meaning and sense of purpose is the cognitive evaluation of the extent to which people feel their life matters and makes sense. Optimism refers to the extent to which people hold generalized favorable expectancies about their future (122) and is assessed primarily in the sense of meaning and purpose. Optimism and its counterpart, pessimism, reflect personality characteristics that develop relatively early in life (123).

#### Importance of Psychological Well-Being

Positive affect has beneficial effects on the neuroendocrine, autonomic, immune, and inflammatory systems (124, 125). It has been linked to better physical, psychological, and social outcomes. Though most research has been conducted with adults, positive affect during adolescence has been associated with better perceived health status and decreased risk-taking behavior and to predict better relationships, positive work functioning, increased self-worth, and healthy adjustment in adulthood (126, 127). Meaning and purpose has been associated with decreased suicide risk in adults (128). It may also function as a protective factor against adolescent health risk behaviors (129). Optimism can be a source of resiliency for youth and has been found to serve as a protective factor for both experiences that youth typically encounter, such as friendship difficulties, and more significant challenges, such as family dissolution or serious medical conditions (130, 131).

### Ethnic-Racial Socialization

The term ethnic-racial socialization is broadly used to refer to the transmission of information (e.g., history, cultural traditions, pride, foods, native language, stories, what it means to have membership in a group, etc.) about ethnicity and race from adults to children. Some specific areas of research conducted on ethnic-racial socialization include: Understanding how parents prepare children to understand and cope with racial barriers and discrimination; children's exposure to ethnic cultural practices; efforts to instill pride in child's ethnic-racial group; identity achievement; and the tension between affiliation with ethnic-racial in-groups and competing pressures to assimilate (132). Ethnic-racial socialization practices can be divided into two components: (1) Cultural socialization, which emphasizes the ethnic group's culture, history, and heritage and (2) Preparation for bias, which prepares children and adolescents for the experience of societal discrimination (133).

#### Importance of Ethnic-Racial Socialization

The transmission of racial and ethnic information is particularly relevant to minority children due to the unique challenges (e.g., social stratification, negative group stereotypes) and institutional barriers that complicate developmental tasks faced by members of minority groups (132, 134). Numerous studies have found that societal discrimination and devaluation of ethnic minority group members are linked to negative mental health outcomes (132, 135–137). As a result, it is important to understand how best to prepare children for experiences with discrimination.

Ethnic-racial socialization practices are also important for White children and families. One study found a significant, positive relationship between the percent of minority children at a White child's school and the frequency of ethnic-racial socialization practices in the home (138). This suggests that the increase of minority students in schools will be accompanied by more discussions about ethnicity and race by White families.

### Perceived Discrimination

Perceived discrimination has been defined as the subjective experience of receiving unfair treatment relative to others (136, 137, 139). Whereas, perceived discrimination may occur among any disadvantaged social group in terms of an ascribed status, the bulk of empirical work has investigated the effects of subjective experiences of ethnic or racial discrimination (137, 140, 141). For the NCS, we propose measuring perceived discrimination of both the parent and child when he/she reaches adolescence.

#### Importance of Perceived Discrimination

Perceived ethnic and racial discrimination has been found to be related to both physical health problems and mental health problems (136, 140). For example, Flores et al. (140) found that higher levels of perceived discrimination among parents of Mexican origin predicted elevated depressive symptoms and poorer general health, even after accounting for levels of perceived stress. According to Williams and colleagues (137), racial discrimination and morbidity are inversely related. Further, perceived discrimination has been linked to domestic violence and youth violence (141) and has been found to predict declines in academic functioning among African-American youth (142) and lower academic well-being among Latino youth (143).

In addition, parents who perceive that they have experienced racial and ethnic discrimination are more likely to transmit socialization messages to their children that prepare them for racism and bias (133). As discussed in the section on racial-ethnic socialization, research findings on outcomes when children receive these messages are mixed. Some studies have found positive associations, including better academic functioning, higher self-efficacy, and fewer depressive symptoms (144). Other studies have linked preparation for bias to increased perceptions of discrimination and lower academic functioning (144, 145). Hughes and Johnson (146) suggest that an overemphasis on racial barriers may have adverse effects on children's self-efficacy and sense of self-worth.

We recommend measurement of perceived discrimination and ethnic-racial socialization at the same time points to ensure capture of both positive and negative experiences related to ethnicity and race. Further, everyday discrimination is conceptualized as a life stressor (137), and we recommend that concurrent levels of general perceived stress should be controlled for in all analyses using this construct. Thus, we recommend that perceived general life stress be measured at the same assessment points as perceived discrimination to enable more rigorous testing of the effects of perceived discrimination unique from general life stress.

### Sexual Orientation: Childhood Gender and Non-conformity, and Attraction, Identity, and Behavior

Sexual orientation is a multidimensional construct that will be measured as childhood gender and non-conformity in early childhood and attraction, identity, and behavior in adolescence. Childhood gender and non-conformity (CGNC) is defined as the extent to which children engage in behaviors that are typical of children of the same biological sex. It is measured by ratings of toy and activity preference, imagined roles, and preferred playmates. CGNC can be assessed in early childhood, whereas sexual attraction, identity, and behavior are more readily assessed after puberty, as a person's sexual interests and desires become more salient.

Sexual orientation has three major dimensions (147, 148): (1) Sexual attraction: The sex or gender that someone feels attracted to; (2) Sexual identification: How individuals identify their sexual orientation (i.e., labels such as gay/lesbian, bisexual, heterosexual); and (3) Sexual behavior: The sex of an individual's sex partners. Although these dimensions are correlated, they do not overlap perfectly, especially in adolescence. Each dimension may have differential associations with health outcomes, so it is important to assess all three whenever possible (149).

#### Importance of Sexual Orientation

CGNC has been found to be associated with later sexual orientation (150, 151) and later sex-typed behaviors, such as identification with others of the same gender, contentment with gender, and self-efficacy with gender-typical activities (152). In addition, CGNC may be a risk factor for being a victim of bullying, especially for boys. Moreover, researchers have found that CGNC is related to pre- and post-natal exposure to androgen hormones (153, 154) and environmental neurotoxic chemicals, like polychlorinated biphenyl (PCBs), dioxins, and phthalates (155, 156). Measuring CGNC in the NCS would have provided a unique opportunity to prospectively collect such data in a large, community sample, in order to, both explore the relation between CGNC, exposure to hormones, and environmental chemicals, and later sexual orientation, as well as, to examine the range of sex-typed play behaviors in the population.

The percentage of American adults identifying as lesbian, gay, bisexual, or transgender (LGBT) was 4.1% in 2016, up from 3.5% in 2012 (157). Moreover, young adults born between 1980 and 1998 were more than twice as likely to identify as LGBT than other age groups (3.2% for those born between 1965 and 1979; 2.4% for birth years 1946 and 1964; 1.4% for 1913 and 1945). The prevalence of non-heterosexual orientation varies widely depending on which components of the construct are used (158). Self-identification as LGBT is only one aspect of measuring sexual orientation, and measures of sexual attraction and behavior often yield larger estimates (159). For example, one large-scale study in Montreal (*N* = 1951) that used the multidimensional construct described above found that 12% of adolescents endorsed at least one measure of non-heterosexual orientation [e.g., reported gay/lesbian or bisexual identity, same-gender attraction, and/or same-gender sexual behavior; (149)].

For all of the attraction, identity, and behavior measures, we strongly recommend that the wording of the measures be carefully reviewed before the initial administration at the 16–17 year old assessment. Given that the measurement of these constructs is constantly evolving and the terms used to describe these concepts is evolving, it is extremely important that the items be current and use best practices available at the time of assessment.

### Religiosity

Religiosity is a formal and informal set of public and private religious activities and experiences that directly and indirectly affect child development.

#### Importance of Religiosity

Research has demonstrated that higher child religiosity has been shown to be a buffer against the effects of child maltreatment, is positively related to school performance and overall health, and is negatively predictive of delinquency, depression, and substance use in adolescents (160–163). Parents' high levels of religiosity is positively associated with better health, higher levels of education and lower levels of mental health problems and substance abuse in their children (164–167).

### Perceived Stress and Resilience

#### Perceived Stress

Perceived stress is the degree to which situations in an individual's life are appraised as stressful (168). It is thought that one's perception of stress has a greater impact on health than how “objectively” stressful events are (168). Response to perceived stress can be divided into two categories: psychological and somatic. Psychological stress responses are thoughts and feelings about self and the world in the context of environmental or internal challenges. This may include cognitive-perceptual disruption, anger, fear, and controllability/manageability. Somatic experiences are the physical sensations associated with responses to internal or external challenges. Somatic stress responses may include arousal, agitation, pain, sleep problems, and gastrointestinal distress (169, 170).

Adolescence is a time that individuals experience increased stress, in part, due to psychosocial and physiologic challenges (including puberty), changing socio-environmental contexts, and the move toward independence (171).

##### Importance of Perceived Stress

Psychological stress is an important determinant in many chronic health conditions, including cardiovascular disease, cancer, and depression (172–174). Perceived stress may be especially salient for children of racial and ethnic minority groups, given that they may often experience significant life stressors such as higher levels of discrimination and stigma (175). An individual's perception of stress is thought to have a greater health impact than objective levels of stress (168). Perceived stress is also related to an individual's resiliency and coping style, and adolescents are often characterized by heightened perceived stress and maladaptive coping patterns (176).

#### Resilience

Resilience can be defined as doing well in the face of adversity. It can be considered a process of adapting and adjusting to significant sources of stress or trauma, and it is facilitated by strengths and qualities related to the individual, their life, and their environment (177, 178). Luthar and Cicchetti offer a formal definition that can be used to guide research: “Resilience is a dynamic process wherein individuals display positive adaptation despite experiences of significant adversity or trauma. This term does not represent a personality trait or an attribute of the individual. Rather, it is a two-dimensional construct that implies exposure to adversity and the manifestation of positive adjustment outcomes (179, 180).” As UNC sociologist Glen Elder and colleague Robert Crosnoe explain, resilience in youth is complex, and both risk and protective factors vary in differing contexts (181). Resilience is the result of three types of promotive factors interacting with risk factors. Promotive factors may be compensatory (factors that “make up for” the negative effects of risk factors); protective (factors that moderate or reduce the effects of other risk factors); or challenge (exposure to these factors may provide skills or resources for coping (182).

##### Importance of Resilience

Assessing resilience can help pinpoint routes to youths' success in the face of adversity. “Persons scoring higher on the resiliency scale are likely to demonstrate better academic skills; have a higher internal locus of control orientation; have higher self-perceived competence in scholastics, jobs, athletic performance, and friendships; and display a wider range of coping skills than are less resilient peers” (183). In a 2007 review, Ozbay et al. document a series of relationships between social support and positive health outcomes (184).

It is difficult to measure resilience itself because it is a construct that is dynamic with the environment. Therefore, we propose measuring sources of resilience. One can also measure individual constructs (e.g., social cohesion as part of social capital, family belonging and involvement, social competence, temperament, negative reactivity, fearfulness, quality of relationships) that may relate to a differential susceptibility to negative life events. Adding a formal measure of sources of resilience, alongside stressful life events, allows for the determination of how these measures relate to resiliency for individuals.

### Summary

The SEB team of the NCS identified a set of child outcomes and environmental influences whose assessment would contribute to our understanding of child health and development from infancy through adolescence. Our focus was on important outcomes for children growing up in a social environment and on constructs that represent social environmental influences on children's growth, development, and health. At the time of the work, the SEB team also identified measures of constructs that were feasible for the context of and within the constraints of the NCS (see Table 1). Assessment of social-emotional and other dimensions of child health can provide a comprehensive picture of the determinants of positive childhood outcomes.

#### Contributions to Science

This paper identifies social-emotional child health outcomes and social environmental influences that are integral to children's health and development from infancy through adolescence. In addition, it recommends measurement tools to assess these constructs that are suitable for use in large-scale longitudinal studies. We believe the work conducted to select measurement domains for the NCS will be useful for other investigators who are seeking to study children's social-emotional and behavioral health over the life course. Indeed, these recommendations were reviewed by the National Institutes of Health Environmental Influences on Child Health Outcomes (ECHO) research program and many were adopted into their measurement framework (185).

## Data Availability Statement

The data supporting the conclusions of this article will be made available by the authors, without undue reservation.

## Author Contributions

CN, DD, ACa, ACh, HG, EP, CK, JH, and RG drafted specific sections of the manuscript, and reviewed and provided edits to the entire manuscript. All authors contributed to the article and approved the submitted version.

## Conflict of Interest

The authors declare that the research was conducted in the absence of any commercial or financial relationships that could be construed as a potential conflict of interest.
